# Segmental Posthetomy in a Four Stallions Case Series

**DOI:** 10.3390/ani11041145

**Published:** 2021-04-16

**Authors:** Adriana Palozzo, Gianluca Celani, Giulia Guerri, Paola Straticò, Vincenzo Varasano, Lucio Petrizzi

**Affiliations:** Unit of Equine Medicine and Surgery, Veterinary 26 Teaching Hospital, Faculty of Veterinary Medicine of the University of Teramo, 64100 Piano d’Accio Teramo, Italy; apalozzo@unite.it (A.P.); gcelani@unite.it (G.C.); pstratico@unite.it (P.S.); vvarasano@unite.it (V.V.); lpetrizzi@unite.it (L.P.)

**Keywords:** equine, segmental posthetomy, prepuce, preputial fold, penis

## Abstract

**Simple Summary:**

The equine prepuce is characteristic for having two infoldings that allow for substantial accommodation of the penis during detumescence and permit erection. Segmental posthetomy of the equine prepuce is a well described technique indicated for penile sheath injuries or lesions (neoplasms, granulomas, or scar tissue) that do not involve the underlying penile tunics. This surgical technique can be performed with variable extent of excision (i.e., the Adam’s procedure or subtotal posthetomy). The authors describe their experiences and results with different levels of preputial resection for treating four Equidae stallion patients with different preputial/pathologies.

**Abstract:**

Segmental posthetomy, also referred to as circumcision, reefing or posthioplasty, consists of removing a circumferential segment of the internal preputial lamina (internal preputial fold) followed by end-to-end anastomosis of skin edges. The purpose of this case series is to describe the successful outcome of segmental posthetomy for treating different diseases involving the internal or/and external preputial fold, while restoring the normal telescopic function. In this paper, we report the first case of complete degloving injury of the equine penis in the literature (case 1) and describe three different common lesions of the equine prepuce/penis (preputial scar tissue in case 2, preputial sarcoid in case 3 and penile/preputial wound in case 4). The amount of prepuce (safe minimums) that can be removed from a stallion without disrupting the proper telescopic function of the internal/external preputial fold and normal copulatory ability, has not been established. In this case series, all Equidae stallions maintained the telescopic function after preputial surgical resection. However, the surgeon must carefully evaluate every single case, especially when performing the Adam’s procedure.

## 1. Introduction

**Figure 1:**
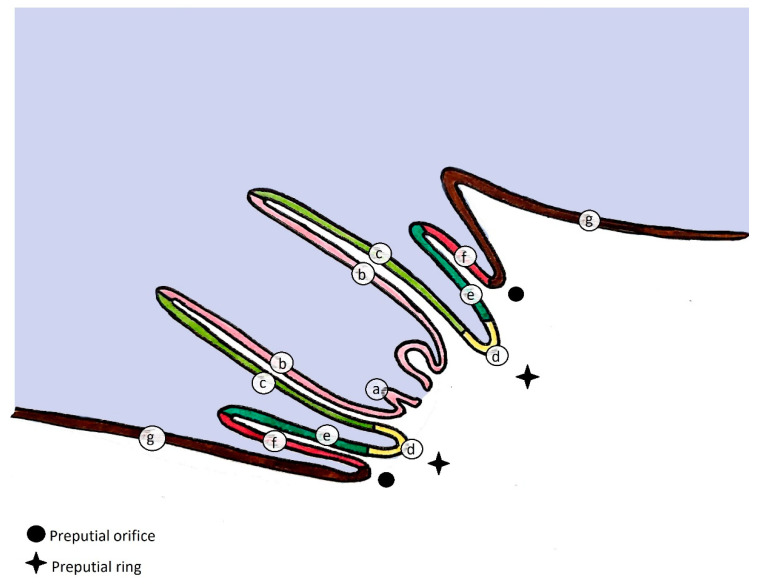
Median-sectional schematic drawing of the equine penis inside the prepuce. (**a**) glans penis; (**b**) free part of the penis; *internal preputial fold (internal lamina):* (**c**) inner lamina of the internal fold, (**d**) preputial ring, (**e**) outer lamina of the internal fold; *external preputial fold (external lamina)*: (**f**) inner lamina of the external fold, (**g**) outer lamina of the external fold.

**Figure 2:**
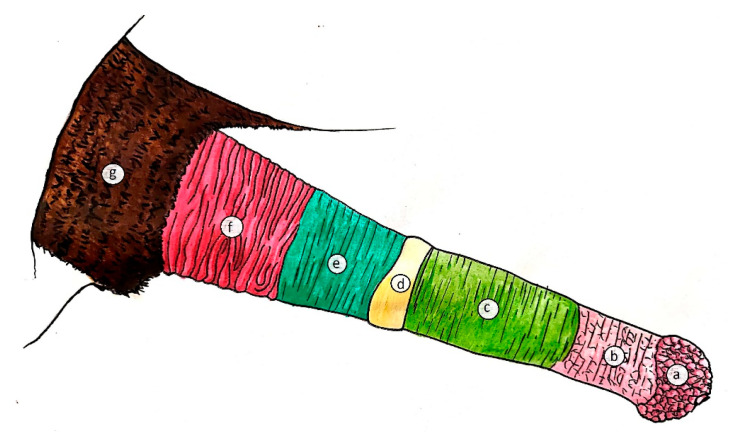
Schematic drawing of the external aspect of the equine penis protruded from the prepuce. (**a**) glans penis; (**b**) free part of the penis; (**c**) inner lamina of the internal fold; (**d**) preputial ring; (**e**) outer lamina of the internal fold; (**f**) inner lamina of the external fold; (**g**) outer lamina of the external fold.

The equine prepuce or penile sheath has a double integumentary fold that covers the free part of the retracted penis: the external preputial fold or external lamina and the internal preputial fold or internal lamina [1]. Each infold consists of an outer and inner lamina or surface or layer (Figure 1 and Figure 2).

Segmental posthetomy, also referred to as “circumcision”, “reefing” or posthioplasty [2], consists of the removal of a circumferential segment of the internal preputial lamina (internal preputial fold) followed by end-to-end anastomosis of skin edges [1].

This surgical technique is recommended for removing granulomas, scar tissue or chronic thickening of the preputial membrane that prevents retraction of the penis [3]. It is also indicated for the removal of neoplasms on the preputial tissue without the involvement of the underlying penile tunic. Extensive segmental posthetomy (i.e., the Adam’s procedure) may be used to maintain a paralyzed penis within the external preputial lamina (external preputial fold) [1].

The aim of this case series is to describe the successful use of segmental posthetomy in four stallions for the treatment of different diseases involving the internal and/or external fold of the prepuce, while preserving the normal telescopic function.

## 2. Clinical Cases

### 2.1. Histories and Clinical Examination

All clinical cases were referred to the Veterinary Teaching Hospital of the University of Teramo (Teramo, Italy).

#### 2.1.1. Case 1

**Figure 3:**
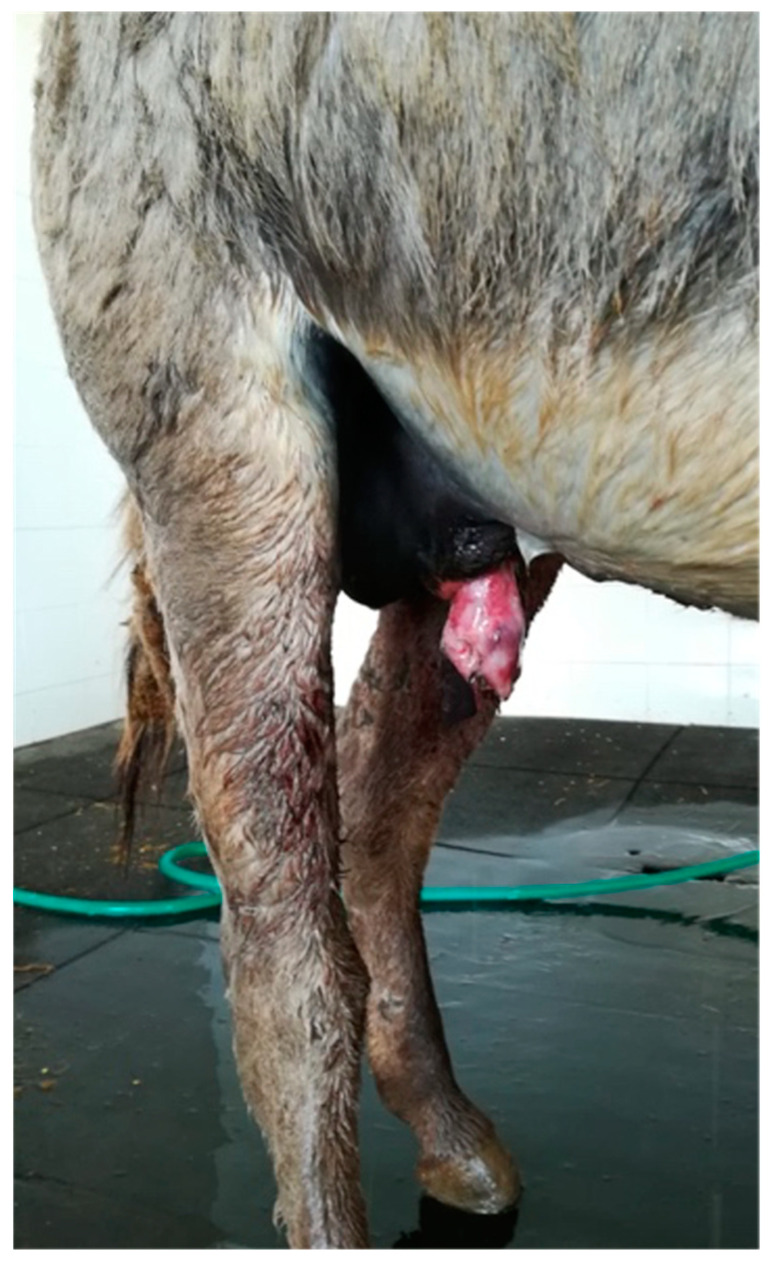
Appearance of the preputial laceration on admission (clinical case 1). The underlying penile tunics were not involved.

A 4-year-old Sardinia donkey stallion was referred with a history of injury of the penis of 4 h duration approximately. A complete circumferential avulsion of the integument at the level where the inner lamina of the external preputial fold inserts on the preputial orifice was detected at physical examination (Figure 3).

#### 2.1.2. Case 2

**Figure 4:**
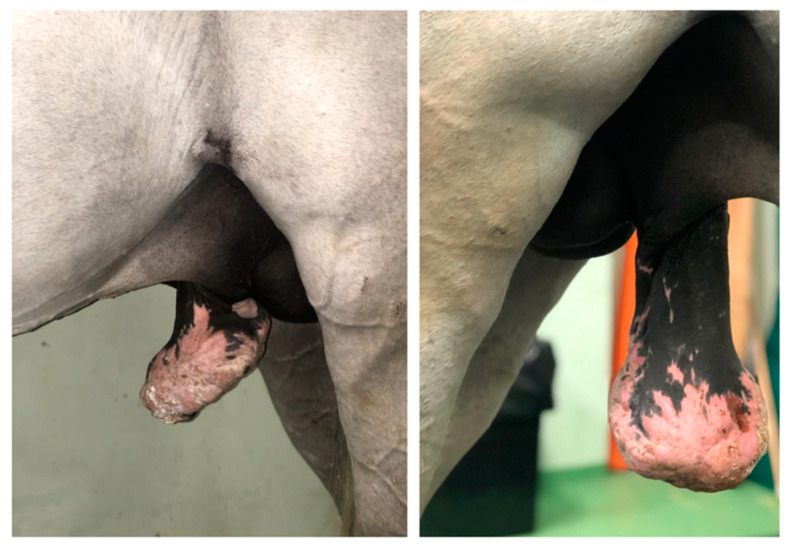
Appearance of the phimosis caused by preputial mass on admission (clinical case 2).

A 14-year-old Pura Raza Espanola (PRE) stallion was admitted with a history of a wide mass against its penile sheath of 6 months duration. On admission, the physical examination of the external reproductive tract highlighted the presence of phimosis, caused by a firm circumferential swelling at the level of the outer lamina of the internal preputial fold and the preputial ring (Figure 4).

**Figure 5:**
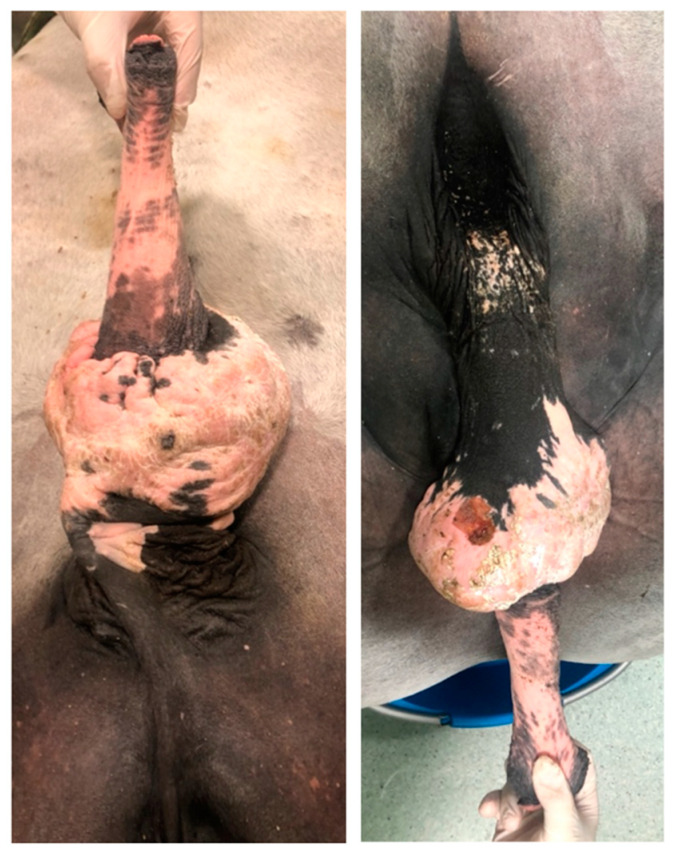
Appearance of the preputial mass after traction on the free portion of the penis, the horse was unable to protrude the penis, through a scarred outer lamina of the internal preputial fold and preputial ring (clinical case 2).

By administering xylazine (0.5 mg/kg intravenously (IV)) and applying traction, the free portion of the penis could be extracted and extended through the acquired stenosis of the preputial ring, and the inner lamina of the internal preputial fold could be examined, to assess that it was not involved (Figure 5).

#### 2.1.3. Case 3

**Figure 6:**
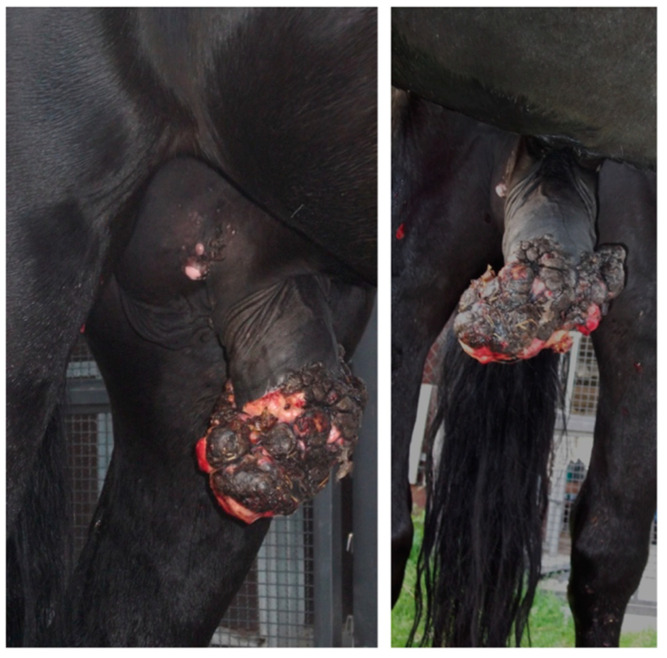
Appearance of the preputial mass on admission (clinical case 3).

**Figure 7:**
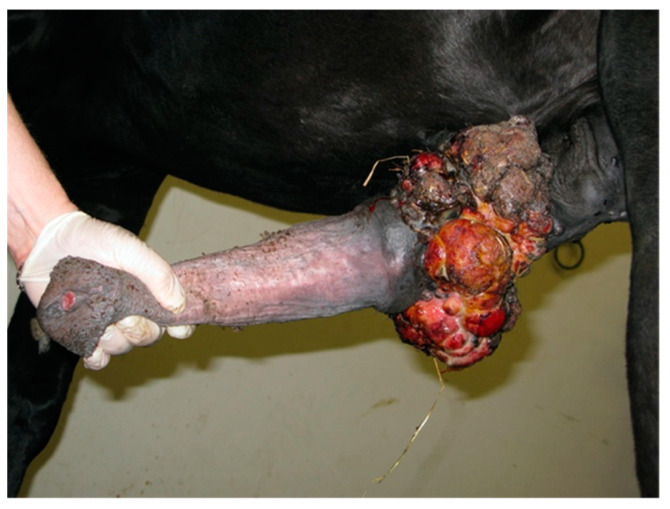
Appearance of the preputial mass after traction on the free portion of the penis (clinical case 3).

A 5-year-old Murgese stallion was referred with a mass over his penile sheath. Physical examination of the external reproductive tract showed a hyperkeratotic proliferative-verrucous neoformation, with ulcerated and infected tissue, surrounding the outer lamina of the internal preputial fold and the preputial ring (Figure 6 and Figure 7). There were other smaller neoformations around the inguinal region. No evidence of regional lymph node involvement was detected externally and per rectum, nor by palpation or ultrasonography.

#### 2.1.4. Case 4

**Figure 8:**
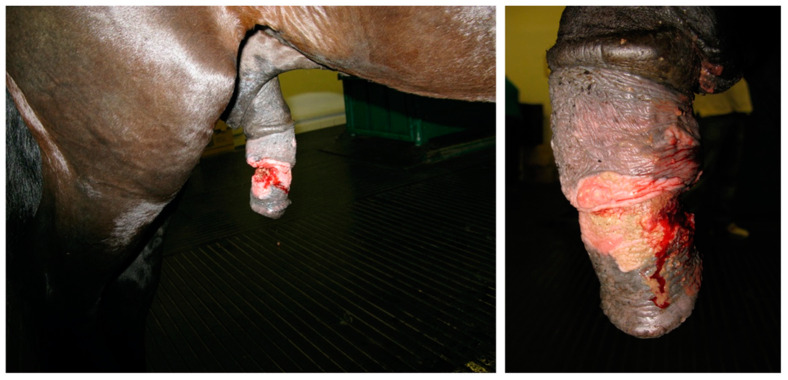
Penile/preputial wound on admission (clinical case 4).

**Figure 9:**
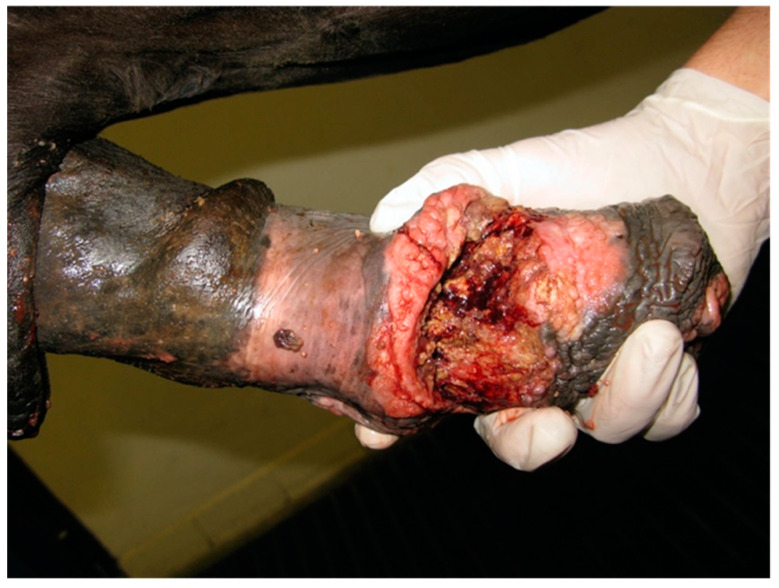
Appearance of the penile/preputial wound at the external physical examination (clinical case 4).

A 16-year-old Pura Raza Espanola (PRE) stallion was referred with a history of injury of the penis of approximately 2 months duration. On admission, a necrotic and ulcerated wound (3–4 cm deep) that involved skin, subcutis and part of the bulbo-spongiosus muscle between the free portion of the penile body and the inner lamina of the preputial fold was detected at physical examination (Figure 8 and Figure 9); the urethra was not involved.

### 2.2. Surgery

#### 2.2.1. Premedication and Preparation of the Patient

In all cases the animals were premedicated with acepromazine (30 μg/kg IM) and sedated with medetomidine (7 μg/kg IV); general anesthesia was induced with ketamine (2.2 mg/kg IV) and diazepam (0.06 mg/kg IV) and maintained with isoflurane and a constant rate infusion of medetomidine (3.5 μg/kg/min IV); all equines were positioned in dorsal recumbency.

Perioperative treatment included the intravenous administration of cefazolin (10 mg/kg EV *bis in die* (BID)), gentamicin (8 mg/kg IV *semel in die* (SID)) and phenylbutazone (2.2 mg/kg IV BID).

After catheterization of the urethra, the penis was extended by traction with a loop of gauze around the *collum glandis*; the glans and the proximal free part of the penis were covered in a sterile bandage, a latex tourniquet was placed at the base of the penile shaft proximal to the preputial ring.

#### 2.2.2. Surgical Treatment

##### Case 1

**Figure 10:**
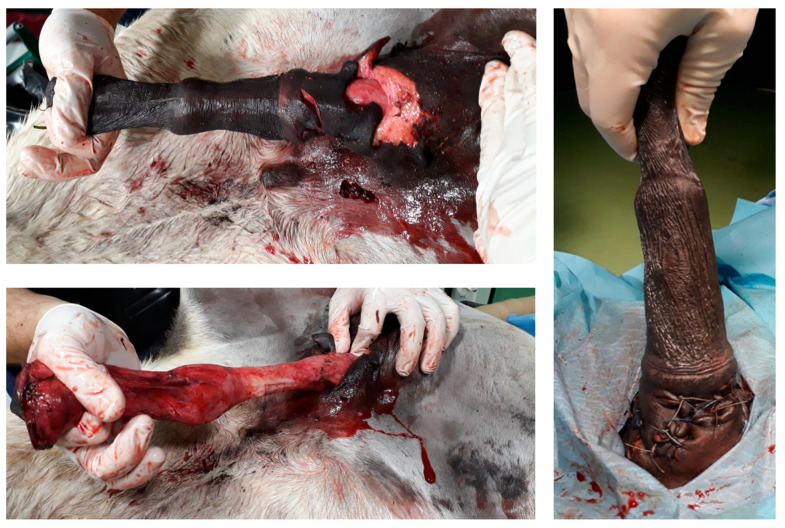
Complete degloving injury of the penis and primary surgical closure (clinical case 1).

At first instance, cleansing, debridement, and primary surgical closure with the animal under general anesthesia using continuous infusion total intravenous anesthesia (triple-drip) was attempted (Figure 10).

**Figure 11:**
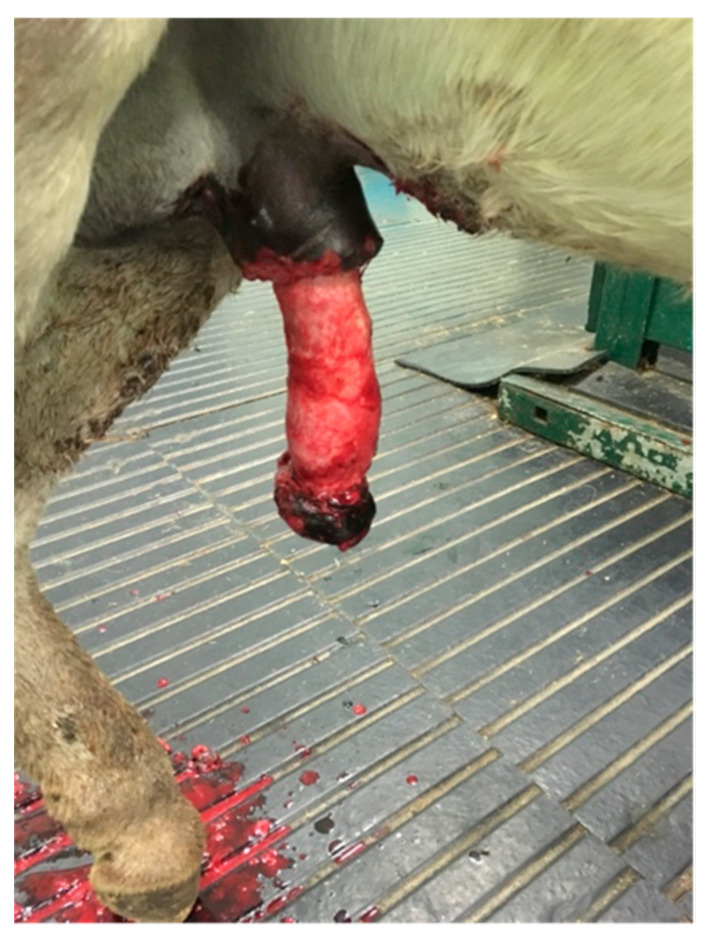
Failure of primary closure on the 12th day after surgical repair (clinical case 1).

**Figure 12:**
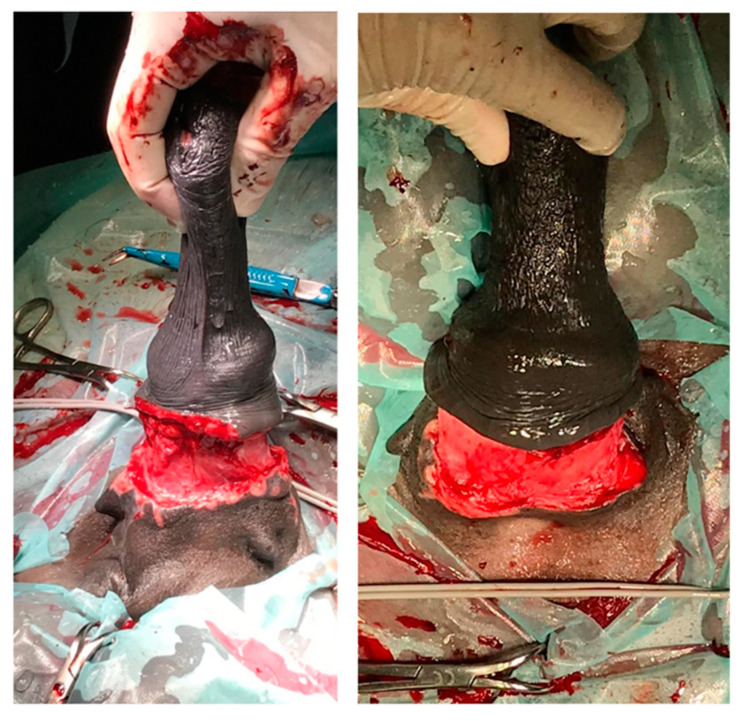
Subtotal preputial resection (clinical case 1).

On day 12 from the first surgery, failure of the primary closure occurred (Figure 11), and a subtotal circumferential preputial resection (inner lamina of the external fold, outer lamina of the internal fold, preputial ring and proximal portion of the inner lamina of the internal fold) (Figure 12) with the donkey under general anesthesia was performed.

**Figure 13:**
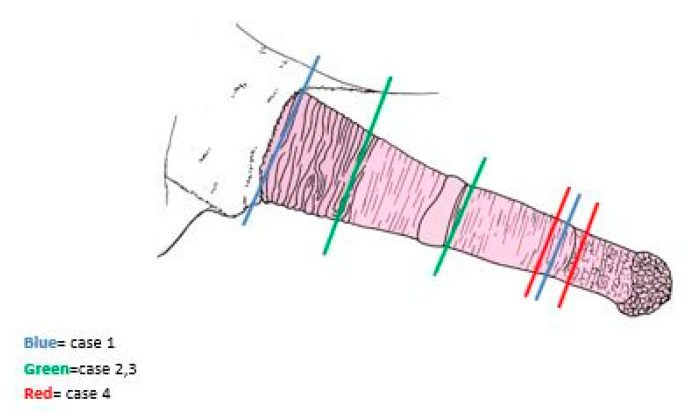
Levels of incisions of segmental posthetomy.

Distally and proximally to the lesion, two parallel incisions, through the preputial epithelium were made (Figure 13-Blue); the distal circular incision was made at the limit between the inner lamina of the internal fold and the free portion of the penile body, and the proximal incision at the level of the external preputial orifice.

**Figure 14:**
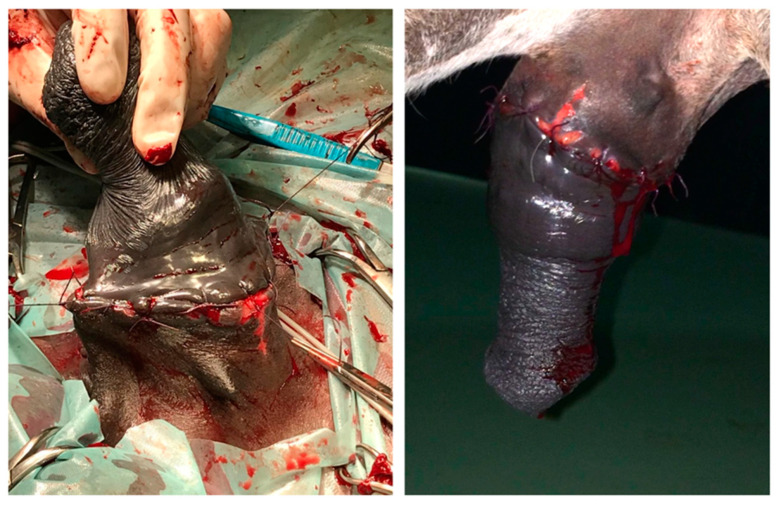
Extensive segmental posthetomy (clinical case 1).

The integument between the incisions was excised. The subcutis and the skin were sutured with a combination of no. 2-0 and 0 sutures (absorbable monofilament material, Biosyn™, Medtronic, Dublin, Ireland) in a simple interrupted pattern, in two different layers (Figure 14).

##### Case 2

A circumferential preputial resection (outer lamina of the internal fold and preputial ring) and anastomosis with the horse under general anesthesia were performed.

Distally and proximally to the lesion, two parallel incisions, through the preputial epithelium were made (Figure 13-Green). The distal circular incision was made at the limit between the inner lamina of the internal fold and the preputial ring, and the proximal incision at the limit between the outer lamina of the internal fold and inner lamina of the external fold.

**Figure 15:**
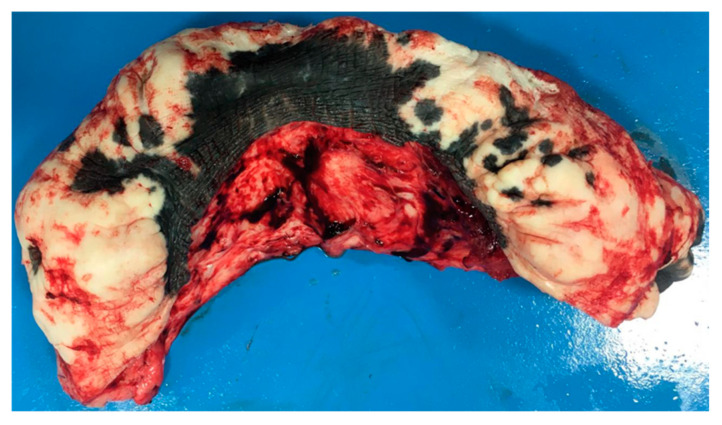
Appearance of the preputial mass after resection (clinical case 2).

The excised mass measured 18 × 10 cm and weighed 560 gr (Figure 15).

The integument between the incisions was excised. The subcutis and the skin were sutured with a combination of no. 2-0 and 0 sutures (absorbable monofilament material, Biosyn™, Medtronic, Dublin, Ireland) in a simple interrupted pattern, in two different layers.

##### Case 3

A circumferential preputial resection (outer lamina of the internal fold and preputial ring) and anastomosis with the horse under general anesthesia were carried out.

Distally and proximally to the lesion, two parallel incisions, through the preputial epithelium were made (Figure 13-Green). The distal circular incision was made at the limit between the inner lamina of the internal fold and the preputial ring, and the proximal incision at the limit between the outer lamina of the internal fold and inner lamina of the external fold.

**Figure 16:**
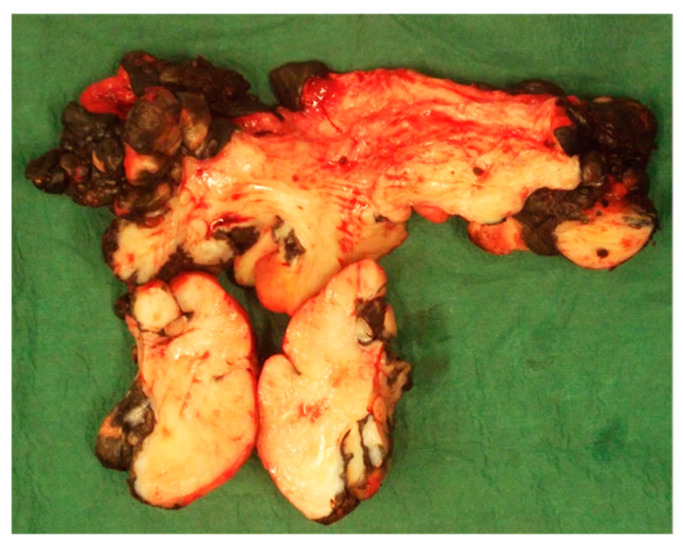
Appearance of the preputial mass after resection (clinical case 3).

The excised mass measured 22 × 11 cm and weighed 600 gr (Figure 16).

The integument between the incisions was excised. The subcutis and the skin were sutured with an absorbable monofilament material no. 2-0 or 0 (Biosyn™, Medtronic, Dublin, Ireland) in a simple interrupted pattern, in two different layers.

##### Case 4

A debridement of the necrotic and ulcerated penile wound of the bulbo-spongiosus muscle between the free portion of the penile body and the inner lamina of the preputial fold was performed; the bulbo-spongiosus muscle defect was apposed by simple interrupted sutures of a no. 2-0 absorbable monofilament material (Biosyn™, Medtronic, Dublin, Ireland).

**Figure 17:**
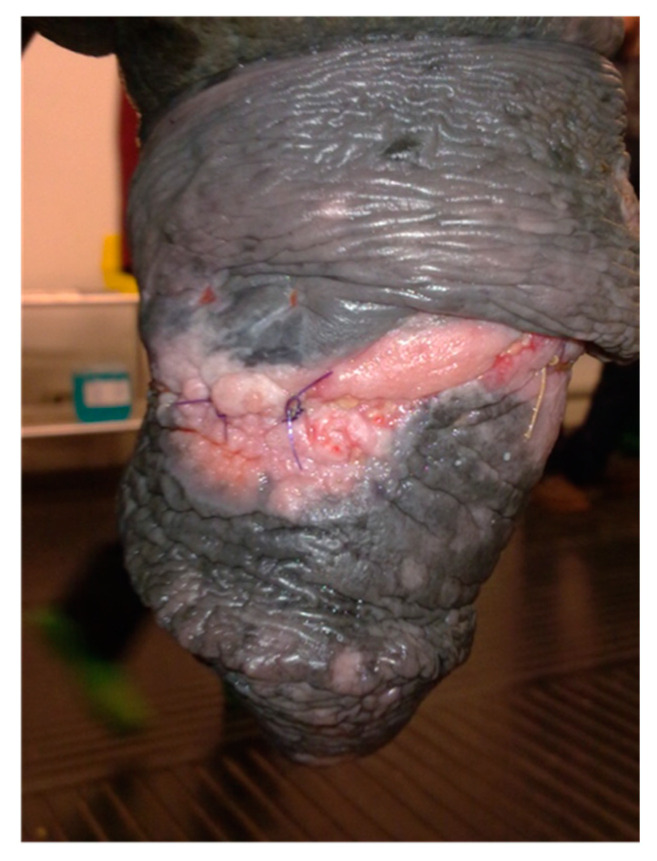
Penile/preputial wound after segmental posthetomy and reconstruction (clinical case 4).

A limited circumferential preputial resection (distal portion of the inner lamina of the internal fold) and reconstruction with the horse under general anesthesia were performed (Figure 17). Distally and proximally to the lesion, two parallel incisions, through the preputial epithelium were made (Figure 13-Red). The distal circular incision was made on the free portion of the penile body, and the proximal incision in the distal portion of the internal lamina of the internal fold.

The integument between the incisions was excised. The subcutis and the skin were sutured with an absorbable monofilament material no. 2-0 or 0 (Biosyn™, Medtronic, Dublin, Ireland) in a simple interrupted pattern, in two different layers.

### 2.3. Post-Operative Care and Follow-Up

Follow-up was obtained via telephone interview with owners and referring veterinarians.

#### 2.3.1. Case 1

Post-operative management included the administration of antimicrobial therapy (cefazolin 10 mg/kg IV BID; gentamicin 8 mg/kg IV SID) for 5 days, anti-inflammatory therapy (suxibuzone 3.3 mg/kg *per os* (PO) SID) for 3 days and application around the surgical site with Hypermix^®^ (RI.MOS. Srl, Mirandola (MO), Italy.) The donkey appeared comfortable when it shafted out to urinate and had normal penile erections. He was discharged 35 days after the second surgical procedure with the preputial integument wound almost completely healed.

Follow-up consultation after 2 years verified that the donkey returned to pre-injury functional status, but he was not used for breeding.

#### 2.3.2. Case 2

Post-operative management included the administration of antimicrobial therapy (cefazolin 10 mg/kg IV BID; gentamicin 8 mg/kg EV SID) for 5 days, anti-inflammatory therapy (suxibuzone 3.3 mg/kg PO SID) for 3 days and application around the surgical site with Reparil^®^ (Rottapharm Madaus, Monza, Italy) The horse appeared comfortable when it shafted out to urinate, and it was discharged 7 days after the surgery. Complications included a mild subcutaneous oedema/hematoma and a mild colic syndrome (within 12 h of anesthesia), which resolved with medical treatment consisting of a single administration of flunixin meglumine 1.1 mg/kg IV.

Follow-up consultation after 6 months verified that the horse returned to work. A breeding career had not been planned for this stallion yet, however the stallion had a normal penile erection.

#### 2.3.3. Case 3

Post-operative management included the administration of antimicrobial therapy (cefazolin 10 mg/kg IV BID; gentamicin 8 mg/kg IV SID) for 5 days and anti-inflammatory therapy (suxibuzone 3.3 mg/kg PO SID) for 3 days. Complications included a mild colic syndrome, which resolved with medical treatment in the following days, and a partial suture dehiscence, which healed by second-intention healing with minimal granulation tissue.

No recurrences occurred over the following 2 years and the stallion was regularly employed for reproduction.

#### 2.3.4. Case 4

Post-operative management included the administration of antimicrobial therapy (cefazolin 10 mg/kg IV BID; gentamicin 8 mg/kg IV SID) for 5 days, and anti-inflammatory therapy (suxibuzone 3.3 mg/kg PO SID) for 3 days.

Recognized post-operative complications were a mild abdominal pain (within 24 h of anesthesia), which resolved with a single administration of flunixin meglumine 1.1 mg/kg IV SID and a partial suture dehiscence, which healed by secondary intention healing. The horse was discharged 10 days after the surgery.

The long-term follow-up reported that the horse, 4 years after the removal of the mass, was in good clinical condition and returned to show jumping competitions, but had not yet been used for breeding; however, he appeared comfortable when urinating and had normal penile erections.

### 2.4. Histopathology

The resected portions of tissue were submitted for histopathology in cases 2–4. Histopathological results are detailed in Table 1.

## 3. Discussion

Circumferential or extensive lesions of the prepuce, that could not be treated with a simple excision, were removed surgically through a more or less extensive posthetomy. Lesions with varying etiology and different extension were treated with success, without recurrences, and with preservation of the telescopic function.

Traumatic injuries of the penis and prepuce occur during breeding when attempting to mate across the fence or from a variety of sources [4,5,6]. There are no reports in veterinary literature describing a complete degloving injury of the equine penis as in the donkey of case 1.

Cutaneous habronemiasis is a granulomatous disease caused by larval infection of *Habronema*. The preputial ring and urethral process are the most commonly involved genital sites [1]. Habronemiasis was the suspected diagnosis of case 2 and 3, but histopathology only confirmed the presence of Habronema larvae in case 3.

The outer lamina of the internal fold was involved in case 3, while the outer lamina of the internal fold and the preputial ring were involved in case 2. A successful extensive posthetomy was performed in case 2 and the horse returned to his intended use.

In case 3, a diagnosis of sarcoid was made histologically. Sarcoid is the most common tumor of the horse and affects horses of all ages. The clinical appearance of this tumor ranges from small single nodules to very aggressive and extensive involvement of the penis and inner lamina of the internal preputial fold [7]. In case 3, the sarcoid involved the outer lamina of the internal fold but not the inner lamina. There were other smaller neoformations around the inguinal region.

In the literature, there are no treatments for equine sarcoids that are accepted as the gold standard [7]. Approximately 40% of affected horses have more than one lesion and up to 50% of horses may have recurrence of the tumor after surgical excision [8,9].

In case 3, segmental posthetomy represented a viable treatment for a large sarcoid located on the outer lamina of the internal fold. A 2-year follow-up proved that no recurrence occurred, and the stallion was regularly employed for reproduction.

Squamous cell carcinoma (SCC) is the most common neoplasm of the penis and prepuce. According to the literature, geldings develop squamous cell carcinomas more often than stallions, and horses with nonpigmented genitalia are more commonly affected than those with pigmented genitalia [10]. Conversely, the horse (case 4) with a squamous cell carcinoma of the free part of the penis was a stallion with pigmented genitalia. The choice of therapy for SCC, depends primarily on the size and the site of the lesion, and the presence of metastases. A standardized algorithm for diagnosis and treatment for penile and preputial SCC in the horse, and a Classification System were developed by Van den Top [11,12].

The histological examination of the resected tissue (case 4) highlighted a grade I SCC (well-differentiated with numerous dyskeratotic cells and prominent keratin pearls, and intercellular bridges) that invaded the penile epithelium and subepithelial tissue. According to the Van den Top algorithm, posthioplasty or local excision were possible treatments, however the choice of therapy for case 4 was not based on this classification because the stallion had a traumatic injury history, and it was treated with the aim of preserving the function of the external genitalia. Despite that the exeresis was not performed as in a case of neoplasm, histopathology showed 2 cm of clean margins.

For such lesions the prognosis is guarded since recurrences and new lesions are common [13]. After excision the outcome is also correlated with the histologic classification: the treatment of moderate to poorly differentiated SCC was unsuccessful in 42.9% horses for grade 2 and 66.7% horses for grade 3 lesions, and in 30.8% horses for grade 1 tumors with well-differentiated cells [10]. Case 4 had a positive outcome and 4-year follow-up proved that no recurrence occurred.

Depending on the nature and extent of the lesion, the amount of prepuce removed in this case series was variable. More specifically, the distal portion of the inner lamina of the internal fold (case 4), the outer lamina of the internal fold and preputial ring (case 2, 3), or the inner lamina of the external fold, outer lamina of the internal fold, preputial ring and proximal portion of the inner lamina of the internal fold (case 1) were removed.

Partial suture dehiscence (case 3, 4) can be managed by second intention healing without compromising the cosmetic and functional result.

Post anesthetic colic (PAC) was recognized within 12 h (case 2) and within 24 h after anesthesia (case 4). In the literature, PAC is also correlated with nonabdominal procedures [14]; the most commonly diagnosed cause of colic is impaction, which responds to medical therapy or no treatment. Medical treatment consisting in a single administration of flunixin meglumine 1.1 mg/kg IV was utilized in case 2 and 4 with a positive response and without extending the anti-inflammatory therapy.

In veterinary literature, posthetomy is suggested for removing the internal preputial lamina [1,15,16,17]. A more extensive surgery (Adam’s procedure), with a distal circumferential incision at the level where the internal preputial lamina inserts on the free body of the penis and a proximal circumferential incision close to the preputial orifice, was described, with the aim of maintaining a paralyzed penis within the external lamina of the prepuce. During this procedure, the surgeon is faced with the challenging task of suturing two different diameter incisions. To accomplish this, the length of the proximal circumferential incision can be decreased by removing two triangles of epithelium from the internal lamina proximal to the posthetomy. Despite the extensive segmental posthetomy that was performed in case 1, the length of the proximal circumferential incision was not decreased, and a positive cosmetic and functional result was obtained.

## 4. Conclusions

The amount of prepuce that can be removed from a stallion without disrupting proper telescopic function of the internal preputial lamina and normal copulatory ability has not been established. In this case series the animals were all Equidae stallions that preserved telescopic function and had normal penile erections after surgery. To our knowledge, this is the only report of segmental posthetomy in stallions.

## Figures and Tables

**Table 1:** Histopathology.

Case	Histopathological Results
2	Fibrotic, sclerotic connective tissue, with mild perivascular inflammatory infiltrates, with the presence of numerous eosinophilic granulocytes; suspected habronemiasis
3	Equine sarcoid; PCR positive for Habronema microstome
4	Squamous cell carcinoma (SCC)

## Data Availability

Data sharing not applicable. No new data were created or analyzed in this study. Data sharing is not applicable to this article.
